# Dataset for concurrent echosounder and ADCP measurements at a tidal energy candidate site in Australia

**DOI:** 10.1016/j.dib.2020.105873

**Published:** 2020-06-17

**Authors:** Constantin Scherelis, Irene Penesis, Mark A. Hemer, Remo Cossu, Jeffrey T. Wright

**Affiliations:** a Australian Maritime College, University of Tasmania, Newnham, TAS, 7248, Australia; b CSIRO Oceans and Atmosphere, Hobart, TAS, 7001, Australia; c School of Civil Engineering, University of Queensland, St Lucia, QLD, 4072, Australia; d Institute of Marine and Antarctic Studies, University of Tasmania, Hobart, TAS, 7001, Australia

**Keywords:** Hydroacoustic dataset, AZFP, ADCP, Concurrent measurements, Environmental monitoring, Tidal resource assessment, Fish aggregations, Australian tidal energy

## Abstract

Interaction uncertainties between tidal energy devices and marine animals have the potential to impede the tidal energy industry as it moves closer towards commercial-scale array installations. Developing standardised environmental impact assessment (EIA) practices would allow for potential impact concerns to the marine environment to be identified and mitigated early during project development. In an effort to help formulate a standardised EIA framework that addresses knowledge gaps in fish-current interactions at tidal energy candidate sites, Scherelis et al. [1] presented a case study for investigating changes in fish aggregations in response to changing environmental conditions including tidal currents at a tidal energy candidate site in Australia prior to turbine installation. Here, we present the dataset utilised for this study titled “Investigating biophysical linkages at tidal energy candidate sites: a case study for combining environmental assessment and resource characterisation” [1]. The dataset includes tidal current information from an Acoustic Doppler Current Profiler (ADCP), volume backscattering measurements from a four-frequency biological echosounder (Acoustic Zooplankton and Fish Profiler – AZFP) as an indicator for fish biomass, and fish aggregation metrics calculated from volume backscatter in post-processing. ADCP and AZFP were installed on a bottom-mounted mooring and engaged in a concurrent sampling plan for ∼2.5 months from December 2018 to February 2019. The mooring was deployed in the Banks Strait, a tidal energy candidate site located in the northeast of Tasmania, Australia, at a location favourable for tidal turbine installations considering current speed, depth, substrate, sediment type and proximity to shore. The ADCP dataset includes current velocity and direction measurements at 1 m vertical and 1-min time intervals. The raw AZFP dataset includes volume backscattering strength collected in 4-s time intervals with a vertical resolution of 0.072 m in raw, and 0.1 m in pre-processed form. Several post-processing steps were implemented to mitigate changes in background noise due to current speed and wind stress, and to isolate acoustic fish returns from remaining scattering sources. Once isolated, volume backscatter containing fish targets underwent post-processing to determine fish aggregation metrics including density, abundance, centre of mass, dispersion,% water column occupied, evenness, and index for aggregation. Each aggregation metric was then binned by minute matched with corresponding environmental conditions for current speed, shear, temperature, diel stage, and tidal stage. Raw and processed datasets for the AZFP and ADCP are provided. Post-processed data includes the derived fish aggregation metrics along with corresponding environmental conditions. The described datasets are freely available on the Australian Ocean Data Network (AODN).

Specifications table

**Table:** 

Subject	Oceanography
Specific subject area	Environmental impact assessment and appraisal for tidal energy
Type of data	Fish aggregation metrics derived from volume backscattering (S_v_) measurements. Current speed and direction measurements.
How data were acquired	Data were acquired with a mooring deployment housing a 38–67–125–200 kHz Acoustic Zooplankton and Fish Profiler (AZFP) from ASL Environmental Sciences and a Signature 500 AD2CP from Nortek.
Data format	Raw Pre-processed Post-processed
Parameters for data collection	The mooring deployment site was selected based on favourable traits for tidal energy turbine development including current speed (up to 2.2 m/s), depth (∼29 m), substrate (sand-gravel), even sea-bottom, and proximity to an existing power grid on shore. Deployment duration was selected to sample several tidal cycles.
Description of data collection	A bottom-mounted mooring was refitted to house a four-frequency biological echosounder (38–67–125–200 kHz) and an ADCP (500 kHz). Instruments were programmed for concurrent data collection with the biological echosounder sampling at 0.25 Hz and the ADCP at 1 Hz over ∼2.5 months.
Data source location	Tidal energy candidate site in the Banks Strait, located between Clark Island and Tasmania, Australia. Deployment depth: 29 m at high tide. GPS location: 40°41′17.3″S; 148°07′21.9″E
Data accessibility	Australian Ocean Data Network (AODN), University of Tasmania, Institute for Marine and Antarctic Studies (IMAS). Available under: https://metadata.imas.utas.edu.au/geonetwork/srv/eng/metadata.show?uuid=5d8d465d-a7a8–4d45-a08b-d89c942244bb (Metadata) https://data.imas.utas.edu.au/attachments/5d8d465d-a7a8–4d45-a08b-d89c942244bb/Scherelis_AZFP_ACDP_Dataset/ (Download page)
Related research article	C. Scherelis, I. Penesis, M.A. Hemer, R. Cossu, J.T. Wright, D. Guihen, Investigating biophysical linkages at tidal energy candidate sites: a case study for combining environmental assessment and resource characterisation, Renewable Energy 159, 2020, 399-413.

## Value of the data

• This dataset was generated to survey interactions between tidal currents and fish at a tidal energy candidate site in Australia. To understand how tidal turbines could potentially interact with fish, it is imperative to establish a baseline for fish-current interactions to help identify and mitigate potential environmental impact concerns prior to significant site development.
• This dataset can benefit industry, regulators and researchers in the tidal energy field looking to correlate results, develop comparable datasets, or test the transferability of processing and analysis methods on another hydroacoustic dataset collected at a tidal energy candidate site.
• Sharing datasets of EIA studies at tidal energy candidate sites allows for effective monitoring practices to be identified and supports the development of a standardised survey approach that would help ensure low environmental impact potentials as the tidal energy industry advances.
• This long-term hydroacoustic dataset collected in a dynamic marine environment in combination with current speed and direction measurements serves as an example for the types of information that can be acquired by combining tidal energy resource characterisation with environmental monitoring efforts.

## 1 Data description

The dataset presented was collected during tidal energy resource assessment of the Banks Strait tidal energy candidate site, Tasmania, Australia. It includes volume backscatter measurements from an echosounder sampling at four frequencies (38–67–125–200 kHz) as well as current speed and direction measurements from an ADCP (500 kHz). Data were sampled concurrently and are given in raw, pre-processed (e.g. with standard hydroacoustic processing operations applied), and post-processed (e.g. calculated fish aggregation metrics) form. A brief description of each data format is given below. This paper presents the dataset utilised in the related research article titled “Investigating biophysical linkages at tidal energy candidate sites; a case study for combining environmental assessment and resource characterisation” by Scherelis et al. [1].

### 1.1 ADCP

Information about sampling resolution and file type for both raw and processed ADCP data are presented in Table 1.

**Table 1:** Sampling resolution and file types of the ADCP dataset.

Data format	Time interval	Vertical resolution (m)	Sampling range	File type
Raw	1-min	1	23	.mat
Processed	1-min	1	23	.csv, .mat

*Raw* – data files produced by the Signature 500 AD2CP must first be corrected for a variety of environmental factors (e.g. sound absorption, transmission losses, etc.) to arrive at interpretable current speed measurements. As such, raw AD2CP files were first uploaded to Nortek's data processing software *Ocean Contour* to derive interpretable current speed and direction measurements, which were then exported as *.mat* files along with the instrument's metadata. Measurements with insufficient beam correlations (<0.5) were excluded and thus only the first 23 cells (e.g. 23 m) presented viable measurements. Current speed measurements are located in the given structure array of the .mat file under: *(‘filename’).Avg_Data.SpeedENUCorrectedDepthSpeed.*

*Processed – raw* data files compiled and appended for time, current speed and current direction for all 23 depth cells in intervals of 1 m. Depth cells were determined from the instrument's internal pressure sensor.

### 1.2 AZFP

Information about sampling resolution and file type for both raw and processed AZFP data are presented in Table 2.

**Table 2:** Sampling resolution and file types of the AZFP dataset.

Data format	Time interval	Vertical resolution (m)	Sampling range (m)	File type
Raw	4–s	0.072	30	AZFP specific file type from ASL Environmental sciences.
Pre-processed	4–s	0.1	30 (surface interference removed)	.mat and .ecs (transducer properties and calibration settings readable with notepad).

**Fig. 1:**
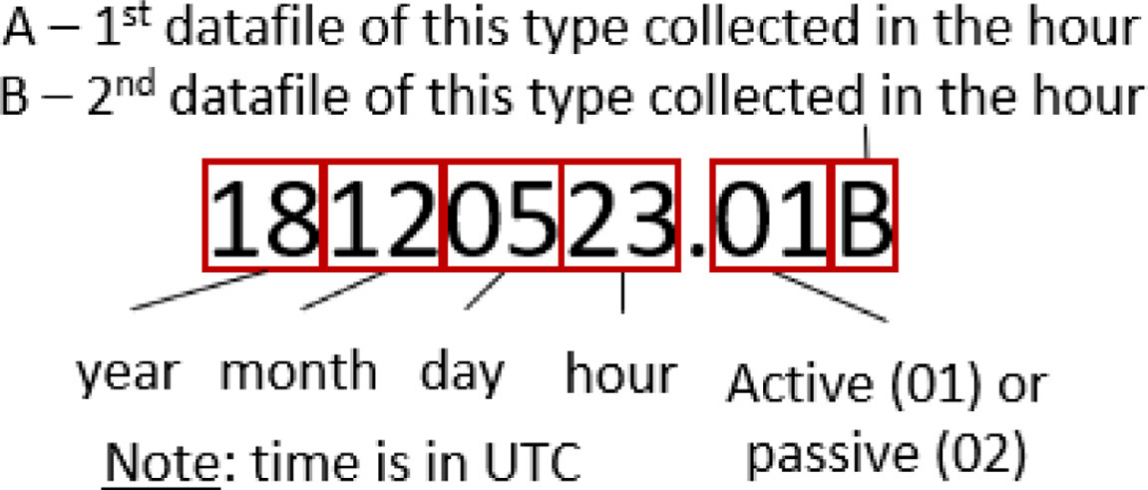
Instructions for interpreting the naming convention for raw data files produced by the AZFP.

*Raw* – Data files produced by the AZFP. Files are readable in the instrument specific software *AZFPLink* by ASL Environmental Sciences, or in specialised hydroacoustics processing software such as Echoview® (10.0, Myriax, Hobart, Australia). Transducer properties are found in the calibration file *AZFPCalibration.ecs*. Given the programmed specification for the instrument to collect 1 min of *passive* data every 30 min, different naming conventions exist to identify *active* and *passive* data. For every hour, a 29-minute data file was created during *active* sampling followed by a 1-minute *passive* sampling period where the instrument did not transmit an active pulse but was still recording in order to assess background and system noise levels. This process was repeated for the second 30-min period of each hour. To identify timing and sampling type of each data file, file names are to be read as presented in Fig. 1.

*Pre-processed* – Volume backscattering strength (S_v_) following adjustments based on the sonar equation, transducer properties (found in *AZFPCalibration.ecs*), and resampling into 0.1 cm vertical cells performed in Echoview®. Timestamps corresponding to each column (i.e. temporal domain) are given in *TimeStamp.mat*, where each row corresponds to the range from the transducer as given in *Range.mat. Time* for the *pre-processed* dataset has been converted from UTC to local time (AEST).

### 1.3 Post-processed

Dataset providing information about fish aggregations in response to prevailing environmental conditions binned by minute. Seven fish aggregation metrics were calculated from volume backscatter corresponding to fish, including density, abundance, centre of mass, dispersion, %-water column occupied, evenness, and index for aggregation. Fish aggregation metrics were then paired with concurrently measured environmental conditions including current speed, shear, temperature, diel stage, and tidal stage. A brief description for each variable in the dataset is given in Table 3.

**Table 3:** Information contained in the post-processed dataset.

*Variable*	*Description*
Time	Minute timestamp of the measurement.
Density (S_v_)	Measure for total fish biomass in the water column.
Abundance (S_a_)	Measure for the abundance of fish scatterers.
Centre of mass (CM)	Mean range of fish from the transducer.
Proportion occupied (P_occ_)	Proportion of cells in the water column that contain fish.
Inertia (I)	Dispersion (or spread) of fish in the water column.
Evenness (EA)	Area occupied if all samples contained the mean fish density.
Index for aggregation (IA)	Measure for the compactness of fish in smaller areas (vs. being evenly distributed in the water column).
Diel stage	Indicates if measurement was taken at night or at day. 1 indicates times after sunrise (e.g. day) and 0 times after sunset (e.g. night).
CSPD	Current speed.
Shear	The absolute mean difference in vertical velocity between the upper and lower layers.
Temperature	Temperature measurement at mooring depth.
Tidal stage	Indicates if measurement was taken during ebb when tidal currents flowed eastward towards the Tasman sea or during flood tide when tidal currents flowed westwards into the Banks Strait. 0 indicates ebb and 1 flood.

Filtering steps applied to isolate volume backscatter corresponding to fish are discussed in Section 2.2. Formula for calculating each metric from the processed volume backscatter values are presented in Table 2 of the related research article [1] along with a more detailed description about the calculated fish aggregation metrics, their implications, as well as the imposed filtering approach.

## 2 Experimental design, materials, and methods

### 2.1 Data collection

The dataset presented was collected as part of the field campaign of the Australian Tidal Energy (AUSTEn) project, a collaborative research project looking to assess the tidal energy potential of Australia and conduct site-specific characterisation studies of high-potential candidate sites [2,3]. One of these tidal energy candidate sites includes the Banks Strait, located between the north-east coast of Tasmania and Clark Island, Australia. See Fig. 1 in the related research article [1] for a map displaying the deployment location and regional tidal currents and bathymetry.

In an effort to help identify best-practices for environmental impact assessment studies of tidal energy sites, monitoring studies were performed pre-turbine installation to survey fish aggregation responses to predominant environmental conditions including tidal currents [1,4]. Studies that inform about interaction potentials between fish and turbines reduce scientific uncertainty and aid in the development of a streamlined permitting process for installing tidal energy devices in high-current regions [5].

This study applied hydroacoustics to investigate interactions between fish and hydrodynamic features (e.g. tidal currents). A bottom-mounted mooring was deployed in 29 m depth (at high tide – 2 m tidal range) in the Banks Strait at a location that exhibited favourable traits for tidal turbine installation including current speeds of up to 2.2 m/s, sand-gravel substrate, even sea-bottom slope, and proximity to an existing power grid on shore (∼11 km) [6,7]. Deployment duration was selected to sample several tidal cycles. The mooring housed a biological echosounder in form of an Acoustic Zooplankton and Fish Profiler (AZFP) to measure volume backscattering strength over four frequencies (38–67–125–200 kHz) and a Nortek Signature 500 AD2CP to measure current speed and direction. Instruments were setup in a concurrent sampling plan with specific collection settings shown in Table 4 and Table 5. Mooring design placed the four AZFP transducers ∼1 m above the sea floor and the Signature 500 AD2CP ∼1.5 m above the sea floor with the instrument reporting a tilt angle of less than 2***°***. See Fig. 2 in the related research article [1] for an image of the deployed mooring showing the placement of each mounted instrument.

**Table 4:** Echosounder collection settings.

Echosounder settings	Acoustic zooplankton and fish profiler (AZFP) transducers			
Frequency	38 kHz	67 kHz	120 kHz	200 kHz
Sampling range (m)	35	35	100	100
Bin size (m)	0.072	0.072	0.072	0.072
Beam angle (°)	12	10	6	6
Sampling rate (hz)	0.25	0.25	0.25	0.25
Pulse duration (τ)	0.5 ms	0.5 ms	0.5 ms	0.5 ms
Sound speed (m/s)	1508	1508	1508	1508

**Table 5:** ADCP collection settings.

Nortek Signature 500 AD2CP					
Sampling range (m)	Bin size (m)	Frequency (kHz)	Averaging interval (s)	Measurement interval (s)	Sound speed (m/s)
23	1	500	59	60	1508

### 2.2 Data processing

Standard processing steps in hydroacoustics involves volume backscatter corrections based on the sonar equation that accounts for time-varied-gain (e.g. transmission and absorption losses), transducer constants and collection parameters [8]. Standard processing steps were performed in Echoview® where system noise determined during the 1-min passive data collection every 30-min was removed and data were resampled into 0.1 m cells. Data were then exported as *.*csv files to undergo statistical processing [9] in Matlab® [9]. Initial data inspection revealed that measurements from the 67 kHz transducer were subject to exceptionally high side-lobe interference and thus disregarded from further processing and analysis steps.

Following pre-processing, a dynamic noise removal approach was implemented to address variable background noise present in the pre-processed dataset. This data filtering component applied customised filter parameters that mitigated the effect of increasing volume backscattering strength with current speed and eliminated the effect of wind-stress induced surface interference [1]. Site-specific filter parameters were chosen based on a sensitivity analysis outlined by Scherelis et al. [1] to achieve comparable background levels during periods of high and low current- and wind speeds. Filter parameters implemented are given in Table 6.

**Table 6:** Filter parameters to mitigate background noise and isolate targets corresponding to fish biomass.

Filter parameters	
Cell statistic window	5 × 15 cells
Blanking distance	2 m for 125 kHz and 200 kHz; 5 m for 38 kHz
Background noise threshold	−81 dB
Filter percentile	46th percentile
Minimum backscatter intensity	−75 dB
Maximum difference in S_v_ allowed among all frequencies	10 dB

Following background noise removal, a dB differencing process was implemented to further isolate acoustic fish returns from other scattering sources in the water column. dB differencing evaluates the frequency response of different scatterers in the water column and eliminates any scatterers whose acoustic signature is highly variable across different acoustic frequencies [10,11]. If volume backscattering strength differed more than 10 dB across the employed sampling frequencies (i.e. 38, 125, and 200 kHz), it would be dismissed as a non-fish target, as fish typically do not exhibit highly variable frequency responses at these frequencies [12,13]. Finally, a minimum acoustic threshold of −75 dB was applied. For a more detailed explanation of the implemented processing steps for each data format (i.e. raw, processed, analysed), please see the ‘Methodology’ section of the related research article [1].

### 2.3 Data post-processing

Once volume backscatter measurements containing fish were further isolated with the applied dB differencing process and −75 dB threshold, fish aggregation metrics were calculated including density, abundance, centre of mass, dispersion, %-water column occupied, evenness, and index for aggregation. This step constitutes the post-processing component. Calculations to derive fish aggregation metrics from filtered volume backscatter measurements (S_v_) were followed according to Urmy et al. [14] and are provided in Table 2 of the related research article [1]. Fish aggregation metrics were binned by minute and paired with concurrently measured environmental parameters including current speed, shear, temperature, diel stage, and tidal stage. This post-processed dataset was then utilised to analyse fish aggregations responses to changing environmental conditions at the tidal energy candidate site in the Banks Strait, Australia.

### 2.4 Dataset limitations

The objective for collecting this dataset was to observe how environmental conditions, especially tidal currents, influence the density and vertical distribution of fish at a tidal energy candidate site. Individual fish species are difficult to parse with the given dataset without prior knowledge about scattering properties of specific fish species or conducting simultaneous fishing activities to ground-truth scattering characteristics to specific fish species or populations. As such, volume backscattering strength presented in the *raw* and *pre-*processed datasets represent backscatter received from all biological and non-biological scatterers within a sampled volume of water. The *post-processed* dataset refers to fish targets of multiple species that satisfied the acoustic filter parameters applied for isolating fish targets specifically.

Filter parameters to isolate fish from other scattering sources must be adjusted based on collection parameters, physical characteristics of the sampling site, and the intended study subject. Ideally, filter parameter outcomes are reviewed manually to evaluate and, if needed, readjust the processing algorithm's parameters for supressing acoustic returns from scatterers that are non-biological and not from the intended study subject (i.e. fish). As such, the *post-processed* dataset carries limitations as a representative for fish aggregation metrics. First, the filtering window (5 × 15 cells) was deemed most appropriate for mitigating background noise, but also eliminates large fish schools that remained in the beam for more than one minute (e.g. 15 horizontal cells) while occupying at least 50 cm (e.g. 5 vertical cells) continuously. Second, fish targets in close proximity to surface interference (i.e. <= 10 cm) caused by protruding air bubbles or turbulence from the surface were also dismissed. Third, fish with no air bladder or small air bladders (e.g. small or juvenile fish) were also dismissed if their backscattering strength was less than −75 dB, as the backscattering strength of a fish is primarily a function of the size of its air bladder [15]. −75 dB represents a fish size of approximately 6 cm according to a general target strength (TS) to target length (TL) conversion formula [16] (following S_v_ to TS conversion). However, this estimate carries limitations as signal strength is known to vary considerably with sampling orientation and morphological features of the fish.

## Declaration of Competing Interest

The authors declare that they have no known competing financial interests or personal relationships which have, or could be perceived to have, influenced the work reported in this article.
